# Concurrent measurement of cerebral hemodynamics and electroencephalography during transcranial direct current stimulation

**DOI:** 10.1117/1.NPh.5.1.015001

**Published:** 2018-01-25

**Authors:** Martina Giovannella, David Ibañez, Clara Gregori-Pla, Michal Kacprzak, Guillem Mitjà, Giulio Ruffini, Turgut Durduran

**Affiliations:** a ICFO-Institut de Ciències Fotòniques, Barcelona Institute of Science and Technology, Castelldefels, Barcelona, Spain; b Starlab, Barcelona, Spain; c Neuroelectrics Barcelona, Barcelona, Spain; d Institució Catalana de Recerca i Estudis Avançats (ICREA), Barcelona, Spain

**Keywords:** transcranial direct current stimulation, diffuse correlation spectroscopy, time-resolved near-infrared spectroscopy

## Abstract

Transcranial direct current stimulation (tDCS) is currently being used for research and treatment of some neurological and neuropsychiatric disorders, as well as for improvement of cognitive functions. In order to better understand cerebral response to the stimulation and to redefine protocols and dosage, its effects must be monitored. To this end, we have used functional diffuse correlation spectroscopy (fDCS) and time-resolved functional near-infrared spectroscopy (TR-fNIRS) together with electroencephalography (EEG) during and after stimulation of the frontal cortex. Twenty subjects participated in two sessions of stimulation with two different polarity montages and twelve also underwent a sham session. Cerebral blood flow and oxyhemoglobin concentration increased during and after active stimulation in the region under the stimulation electrode while deoxyhemoglobin concentration decreased. The EEG spectrum displayed statistically significant power changes across different stimulation sessions in delta (2 to 4 Hz), theta (4 to 8 Hz), and beta (12 to 18 Hz) bands. Results suggest that fDCS and TR-fNIRS can be employed as neuromonitors of the effects of transcranial electrical stimulation and can be used together with EEG.

## 1 Introduction

Transcranial current stimulation (tCS) is a noninvasive form of brain stimulation that applies weak direct electrical currents to the brain through electrodes placed on the scalp. Different types of stimulation are available based on how the current delivered to the brain is modulated.^1^ The most common approach is to hold the current constant during the stimulation period, this is known as transcranial direct current stimulation (tDCS). This method is commonly referred to as tDCS, but in order to avoid confusion with the optical method, we here use DC-tCS. DC-tCS induces bidirectional, polarity-dependent changes in cerebral excitability of humans^2^^,^^3^ and has been shown to modulate cognition in both healthy subjects (improvement of cognitive function) and in patients (reversing the effects of cognitive deficits after a stroke).^4^^,^^5^ Positive effects have also been shown in treatment of depression, chronic and acute pain, Parkinson’s disease, focal epilepsy, and for improving recovery after stroke.^6^^–^^9^ A therapeutic application of this technique is appealing due to its relative low cost, to its demonstrated safety,^10^ and as a substitute for pharmacotherapy, especially for patients with poor drug tolerability.^8^

In order to define protocols of application and to optimize the individual dosage of stimulation, the effects of the stimulation on the cerebral activity must be monitored in real time.^11^^,^^12^ Availability of a concurrent read-out of the stimulation effects could improve its effectiveness and, ultimately, a personalized stimulation driven by the neuromonitor feedback can be imagined.^13^ For this purpose, due to the complex nature of cerebral activity, multimodal monitoring is preferable,^14^^,^^15^ such that different monitors integrated in a single set-up can allow to record the effects at different levels, from neuronal activity, to hemodynamics to systemic.^16^ The neuronal activity is evaluated by electroencephalography (EEG), which measures the synchronous activation of a large population of pyramidal neurons oriented perpendicularly to the scalp. On the other hand, cerebral hemodynamics can be used as a surrogate measure of the cerebral activation assuming that cerebral blood flow (CBF) increases in a region of the brain, where neurons and synapses are activated in order to meet the demand for more energy and that the amount of local increase of blood flow and the changes in oxygen saturation are related to the cellular energy consumption.^17^

Even if different options are available as neuromonitors of hemodynamics, functional near-infrared spectroscopy (fNIRS) appears to be the best suited for this application.^18^ The most accessible and commonly employed fNIRS technology uses continuous-wave sources (CW-fNIRS), where constant intensity light sources are employed to measure oxy- (${\mathrm{HbO}}_{2}$) and deoxyhemoglobin (Hb) concentration changes as surrogate measures of CBF. fNIRS has advantages that are fundamental for this application. In particular, it allows for continuous noninvasive measurements, does not require the immobility of the subject, and is not necessary to change the environment preferable for the stimulation to integrate CW-fNIRS in the protocol.^18^ It has indeed already been used as a monitor during DC-tCS, both in animals^19^ and on humans at rest^20^^–^^22^ and during tasks.^22^^–^^24^ It has also been used together with EEG to monitor DC-tCS^25^ and a device integrating the two monitors has been developed and characterized.^26^^,^^27^ Despite of the successes of these studies, it must be noted that CW-fNIRS does not allow a quantification of absolute value of ${\mathrm{HbO}}_{2}$ and Hb concentration, lacks precision and accuracy,^28^ and does not give a direct measure of CBF, the primary marker of the neurovascular coupling.

In this work, we propose to use time-resolved fNIRS (TR-fNIRS) and functional diffuse correlation spectroscopy (fDCS) as neuromonitors of the brain stimulation. Using pulsed light of few hundreds of picoseconds in width, TR-fNIRS measures absolute values of Hb and ${\mathrm{HbO}}_{2}$ concentration by separating the effects of absorption and scattering. The time information can also be used to explore different behaviors in different layers of the tissue.^29^ On the other hand, fDCS^30^ allows a direct measure of CBF in the microvasculature of the tissue.

Here, fDCS and TR-fNIRS are used together with EEG to monitor cerebral activity before, during, and after 10 min of DC-tCS over the frontal cortex. This area is the most convenient choice for the diffuse optical monitors because of the lack of hair. Since its stimulation has been proven effective for the improvement of various cognitive functions,^31^^–^^38^ for example, working memory enhancement,^39^^–^^42^ and for treatment of depression,^9^^,^^43^^,^^44^ it is also a relevant area to stimulate and study.

## 2 Methods

### 2.1 Transcranial Direct Current Stimulation and Electroencephalography Device

**Fig. 1:**
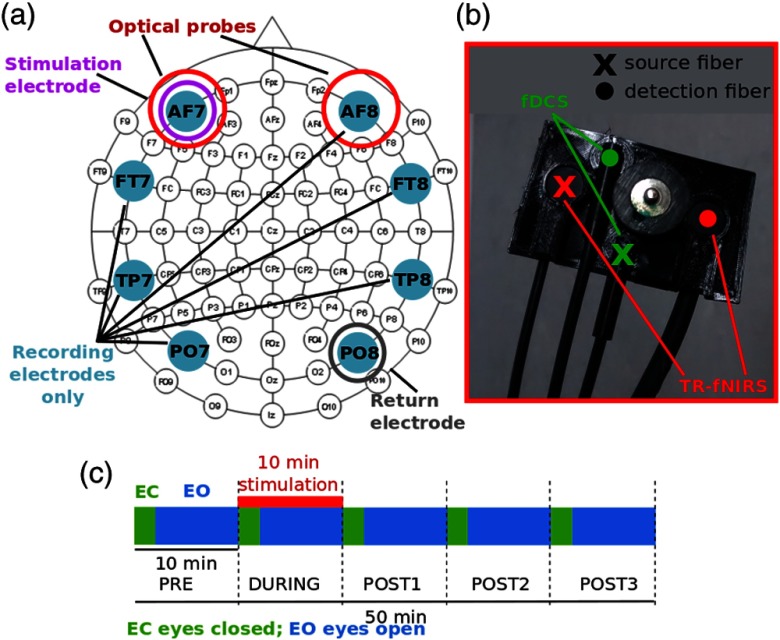
(a) A schematic of the optical probes, stimulating and return electrodes and EEG recording electrodes superimposed on a standard 10–10 EEG system. (b) Image of the integrated probe that could accommodate optical fibers (both for fDCS and TR-fNIRS) and an electrode. (c) Schematic of the protocol that lasted a total of 50 min divided in five 10-min periods. Stimulation was delivered during the second period.

DC-tCS was delivered using Neuroelectrics Starstim^®^ (Neuroelectrics, Barcelona, Spain), which is a research-class eight channel transcranial current stimulator that is also capable of measuring EEG. About 12-mm diameter silver chloride electrodes (Ag/AgCl) with conductive saline gel were used for EEG recording and for stimulation. Eight electrode (AF7, AF8, FT7, FT8, TP7, TP8, PO7, and PO8) locations were chosen, as depicted in Fig. 1(a), according to the 10–10 EEG system.^45^ In particular, the active electrodes during stimulation were AF7 (left frontal lobe) and PO8 (right parieto-occipital lobe). From now on, we will refer to the former (AF7) as the stimulation electrode while to the latter (PO8) as the return electrode. All the eight electrodes functioned as EEG recording electrodes for protocol periods that did not involve stimulation. EEG spectrum was sampled at 500 Hz.

### 2.2 Diffuse Optical Monitors

Two diffuse optical monitors were employed to monitor cerebral hemodynamics during and after the stimulation. A commercial fDCS device (HemoFloMo, HemoPhotonics S.L., Castelldefels, Spain) with four source (785 nm) and eight parallel detector channels was used to measure CBF in the microvasculature. In addition, a prototype of a TR-fNIRS system (TRS-20, Hamamatsu Photonics K.K., Hamamatsu City, Japan) was used for TR-fNIRS. It works at three wavelengths (760, 800, and 830 nm) and has two independent source–detector channels. Hardware triggers allowed the communication between the two optical neuromonitors in order to measure alternately without interfering with each other. The procedure was fully automatized with fDCS defined as the “master.” This means that fDCS triggered the start of the acquisition of TR-fNIRS and then paused the acquisition until a signal was received from TR-fNIRS. Furthermore, the marker signals were introduced by the operator on the fDCS device, which sent a signal to TR-fNIRS in order to have the markers registered in the TR-fNIRS.

Since the measurement of the two devices was sequential, the temporal resolution of the hemodynamics parameters was the sum of the fDCS averaging time (1.5 s) and the TR-fNIRS integration time. The TR-fNIRS averaging time was adjusted between 3 and 4 s for each subject in order to gather enough photons to have three decades between the background noise and the peak of the collected TR-fNIRS distribution of the time-of-flight (DTOF) of photons.

Since fDCS and TR-fNIRS were both meant to probe the area under the corresponding electrode, an integrated probe holder was designed and 3-D printed with a flexible material. As shown in the image in Fig. 1(b), the flexible probe could accommodate fibers for fDCS (source–detector separation 2.5 cm), for TR-fNIRS (source-detector separation 3.7 cm), and an electrode. Two of these probes were placed on the forehead of the subject, one on the left hemisphere in correspondence with AF7, carrying the stimulation electrode, while the other on the right hemisphere in AF8 position with a purely EEG recording electrode. Two of the four sources available for fDCS were used, one for the left and one for the right hemisphere. For each hemisphere, only one detection position was implemented using a bundle of four single-mode fibers probing the same area, i.e., all the eight detector channels were used. The correlation curves collected by four fibers in the same area were averaged in order to improve the signal-to-noise ratio. On the other hand, the two independent source–detector channels of the TR-fNIRS device were used for the two hemispheres.

### 2.3 Protocol

Healthy, right-handed adult subjects were recruited for three sessions of combined stimulation and measurement: anodal [i.e., anode in AF7 (stimulation electrode) cathode in PO8 (return electrode)], cathodal (i.e., cathode in AF7, anode in PO8) stimulation sessions, and a sham session. Such a montage creates an electric field that crosses the brain as confirmed by simulation of the electric field distribution in the brain (data not shown). For half of the subjects, the anodal was the first session, while for the other half cathodal was. Sham stimulation was always given during the third session. All sessions were separated by a wash-out period of at least 1 week. The studies were approved by the ethical committee of Hospital Clinic in Barcelona. Each subject signed an informed consent and the study was conducted according to the principles of the Declaration of Helsinki. Exclusion criteria included the history of neurological and psychiatric conditions and the current use of psychoactive medications.

A schematic of the protocol is presented in Fig. 1(c). The protocol consisted of 50-min measurement sessions divided into five periods. After 10 min of baseline (pre-period), 10 min of stimulation was given (during period), then 30 more minutes were recorded (post1, post2, and post3 periods). During the stimulation, a current of 1 mA (resulting in a current density of $0.88\;{\mathrm{mA}/\mathrm{cm}}^{2}$) was delivered to the brain with a ramp up of 30 s at the beginning and a ramp down of 30 s at the end of the stimulation period. Such a duration for ramp up and down was chosen considering that a shorter one could cause an itching and disturbing sensation for the subject. In the sham session, the current was ramped up to 1 mA in 30 s and then brought back to 0 in 30 s. After 8 min, it was again ramped up and down to simulate the real stimulation sessions. This design gave the subjects the same feeling as a real stimulation because the ramp up and down periods are usually the most noticeable moments. However, the stimulation was too short to have significant, durable effect on the brain activity.

For each 10-min period, the subject was asked to close the eyes for the first 2 min (from 0 to 2, from 10 to 12, 20 to 22, 30 to 32, and 40 to 42 min). During the eyes-open condition, subjects were requested to stare at a screen, where a cross was shown to change color or to rotate at random time (but between every 20 or 30 s) and to press a key when the cross configuration changed in order to maintain the subject gaze and attention to a fixed point.

### 2.4 Data Analysis

For TR-fNIRS analysis, three different approaches were used. (1) First, the so-called differential pathlength factor (DPF) analysis was implemented.^46^ Total count rate and the modified Beer–Lambert’s law were used to calculate the absorption changes and consequently the changes in Hb and ${\mathrm{HbO}}_{2}$ concentration in order to simulate what a typical CW-fNIRS device would have registered. (2) Second, the DTOF of photons collected after propagation in the tissue was fit to the solution of the photon diffusion equation for a semi-infinite homogeneous medium for the reflectance in the time-resolved regime.^47^ A measure of optical properties (absorption and reduced scattering coefficient) for each wavelength was derived and an absolute measure of Hb and ${\mathrm{HbO}}_{2}$ concentrations was calculated. This option gave more accurate results with respect to the DPF analysis because complete information on the tissue optical properties was retrieved. To improve the stability of derived optical properties, the reduced scattering coefficient was kept fixed to the mean of the baseline value (pre-period) during the whole protocol. This approximation assumed that metabolic changes affect only the blood oxygenation but not the tissue scattering properties. (3) The last approach included an analysis of the second central moment (variance) of the DTOF. Changes in the absorption coefficient were derived from changes in the variance.^48^ Through Monte Carlo simulations of photon propagation into the tissue, it was demonstrated that the variance is more sensitive to changes in the deeper layers compared to the intensity (used for DPF analysis), which is more sensitive to the superficial layer. For this reason, we have compared results from DPF and variance analyses.

DCS measurements were analyzed using the solution for the diffusion equation of the electric field autocorrelation function for the semi-infinite medium^30^ to derive the blood flow index (BFI). Optical properties derived from TR-fNIRS were used in the analysis. Once derived, the BFI, a measure of CBF, was normalized to the baseline (first 10 min of acquisition) to obtain a measure of relative CBF (rCBF).

Changes in the EEG patterns induced by the brain stimulation were independently analyzed for eyes open and closed conditions. Pre- and post-stimulation EEG recordings were filtered using a finite impulse response filter of 500 coefficients with cut-off frequencies set to 1 and 20 Hz. Filtered recordings were split into 60-s blocks in which power at delta (2 to 4 Hz), theta (4 to 8 Hz), alpha (8 to 13 Hz), and beta (12 to 18 Hz) bands were calculated. Each 60-s block was further split into 2-s windows with a 50% overlap. After individual epoch detrending, those epochs containing samples larger than 75  μV, considered high amplitude artifacts, were rejected. Relative power at the previously defined frequency bands was calculated via trapezoidal power spectral density integration and was normalized with respect to the full band power (2 to 18 Hz). Power at 60-s blocks was calculated as the average power of artifact-free 2-s epochs.

### 2.5 Statistical Data Analysis

Outlier detection was implemented by analyzing the functional depth,^49^^,^^50^ i.e., the centrality of a given curve within a group of trajectories, with each curve representing the response of cerebral hemodynamics or EEG power. The R package^51^ “fda.usc”^52^ and the R function “Outliergram”^50^ were used as tools for this purpose. The outlier detection was run in the two hemispheres independently for the cerebral hemodynamics and for each electrode and band for the EEG response. A subject was defined as an outlier if it was detected by both methods simultaneously and was, thus, removed from the statistical analysis. If a subject was defined an outlier for ${\mathrm{HbO}}_{2}$ concentration, then it was removed also for Hb concentration, and vice versa.

A linear mixed effects (LME) analysis was performed to test the effect of the stimulation session and time on the cerebral hemodynamics and EEG response using the R package “lme4.”^53^

For cerebral hemodynamics, we treated the two hemispheres independently building an LME model for each of them. To include time, we selected periods of 4 min during which we averaged the response: minute 14 to 19 as during DC-tCS period, minute 24 to 29, 34 to 39, 44 to 49 as post1, post2, and post3 DC-tCS periods, respectively. Response was normalized to the last 4 min of the pre-period; therefore, this period was not introduced in the analysis.

Proceeding to EEG power change, with respect to initial baseline, each band and electrode was evaluated separately, as well as eyes opened and eyes closed periods. Post-periods were introduced in the LME by averaging the response in each of them.

In all the models, the period and the stimulation type (and their interaction) entered as fixed effects. As random effects, the intercepts for subject and the random slope for period on a subject basis were considered. For EEG, only a random intercept was used since the model would not converge if the random slope had also been added. Residual plots were tested for the inspection of deviations from homoscedasticity or normality. Models were built with forward steps following likelihood ratio tests of the model with the effect in question against the model without the effect in question. The Bayesian information criterion parameter was considered to assess whether the effect was improving the model. Stimulation type was the first to be tested. If found significant, the period was added and a new model was tested. If this step was also successful, the interaction between the two was also tested.

Posthoc analysis was performed with R package “lsmeans”^54^ to quantify changes in hemodynamics parameters and in EEG power for each stimulation session and period. To this end, least-square means of the linear model were calculated and the different sessions were compared using the Tukey correction for multiple comparisons. For data visualization, a bootstrap sample was calculated for the time series of cerebral hemodynamics parameter. The bootstrap method is based on consecutive and random resampling of the sample distribution^55^ and was implemented in the R package “fda.usc.”^52^

## 3 Results

A total of 20 healthy subjects (nine female) participated in anodal and cathodal stimulation sessions. Twelve of them came back for a sham session. Technical problems resulted in one subject for TR-fNIRS measurement being discarded for the cathodal session leaving 19 subjects for ${\mathrm{HbO}}_{2}$ and Hb concentration measurements. In addition, one fDCS measurement was discarded during the sham session leaving 11 subjects for flow measurement during sham stimulation. Technical exclusion criteria for EEG, explained in Sec. 2.4, resulted in 20 subjects for anodal, 16 for cathodal, and 9 for the sham session. As explained above, outliers were detected and excluded from the statistical analysis. The number of subjects surviving the outlier detection for EEG and hemodynamics parameters is presented in Tables 1 and 2, respectively.

**Table 1:** The number of subjects for EEG that were used for the statistical analysis for each electrode and for each band, after the technical exclusion criteria and outlier detection.

	Sham				Anodal				Cathodal			
	Alpha	Beta	Delta	Theta	Alpha	Beta	Delta	Theta	Alpha	Beta	Delta	Theta
AF7	9	8	9	8	20	20	19	19	15	15	16	15
AF8	9	9	9	8	20	19	19	19	15	15	16	15
FT7	9	9	9	9	19	19	18	19	16	15	16	14
FT8	9	9	9	8	20	17	19	20	16	15	14	15
PO7	9	9	9	8	19	19	19	19	16	15	16	15
PO8	8	9	9	7	20	19	18	19	15	15	16	15
TP7	9	9	9	8	19	19	19	19	15	14	16	15
TP8	9	9	9	7	18	19	19	18	15	15	16	15

**Table 2:** The number of subjects for hemodynamics parameters that were used for the statistical analysis after the technical exclusion criteria and outlier detection.

	Sham		Anodal		Cathodal	
	Left	Right	Left	Right	Left	Right
CBF	11	11	18	18	20	18
${\mathrm{HbO}}_{2}$ and Hb	12	12	18	19	17	17

**Fig. 2:**
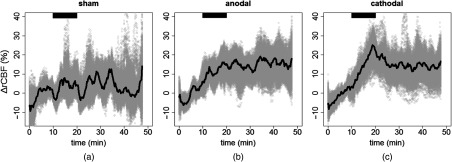
rCBF change during the three types of stimulation sessions on the left hemisphere. (a) Sham session, (b) anodal session, and (c) cathodal session. The thick black line is the average, and the gray area represents the bootstrap sample over all the subjects. The black horizontal bar highlights the stimulation period.

In Fig. 2, the time series of rCBF change, averaged over subjects, is shown together with the bootstrap sample. Only results from the left hemisphere (ipsilateral side, under the stimulation electrode) are shown from the three different stimulation sessions.

When building the LME model from the null one, the stimulation type is found to improve it for blood flow in the left hemisphere, but not in the right one. Therefore, blood flow response depends on the stimulation in the ipsilateral hemisphere, but not in the contralateral one. Period is not an effect that improves the model for either of the hemispheres. Accordingly, we can conclude that blood flow stays constant at the end of the stimulation period (during) and in the post-periods. Posthoc analysis results are summarized in Fig. 6. Blood flow increases significantly from the baseline by 10% [confidence interval (CI): 7% to 14%] for anodal (p<0.001) and 11% (CI: 7% to 15%) for cathodal (p<0.001) stimulation while no increase is seen in the sham session (p=0.07). A comparison between the different sessions revealed a detectable difference between anodal and sham (p=0.008) and cathodal and sham (p=0.003) sessions while no difference is seen between anodal and cathodal sessions.

**Fig. 3:**
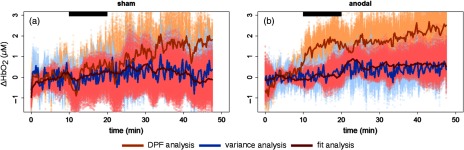
Changes in oxyhemoglobin (${\mathrm{HbO}}_{2}$) concentration during (a) sham and (b) anodal stimulation, in the left hemisphere, according to the three types of analysis considered for TR-fNIRS: orange for DPF analysis, blue for the variance, and red for the fitting analysis. Continuous line is the average of all the subjects, the lighter colored region represents the bootstrap sample with the same color code. The black horizontal bar highlights the stimulation period.

In Fig. 3, the results for the time evolution of ${\mathrm{HbO}}_{2}$ concentration from TR-fNIRS measurements using the three different analysis methods are shown for anodal and sham stimulations for the left (ipsilateral) hemisphere. DPF analysis gives an increase of ${\mathrm{HbO}}_{2}$ concentration starting during the stimulation and persisting after it. Notably, this change is seen both in the anodal and sham sessions. The results obtained with the analysis of variance and by fitting are comparable and show smaller changes than the DPF analysis both in anodal (p<0.001) and sham (p<0.001) sessions.

**Fig. 4:**
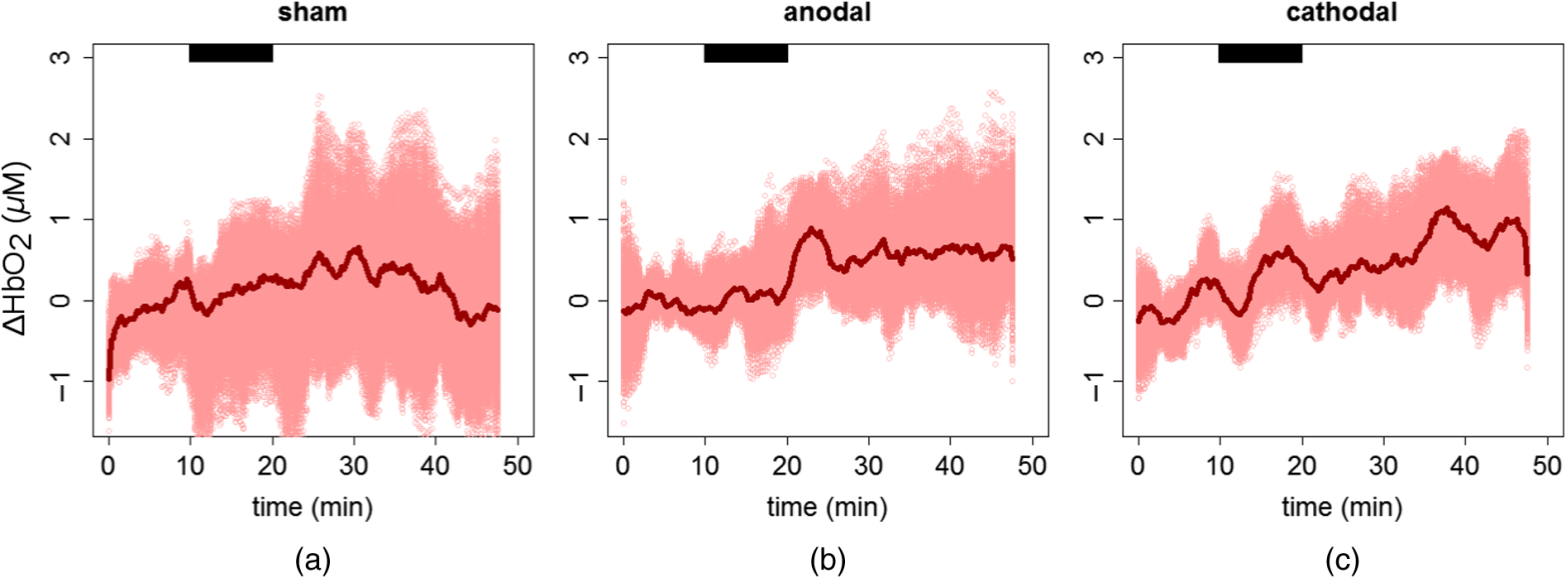
Oxyhemoglobin (${\mathrm{HbO}}_{2}$) concentration during the three types of stimulation sessions on the left hemisphere from the fit analysis. (a) Sham session, (b) anodal session, and (c) cathodal session. The thick line is the average, and the lighter colored region represents the bootstrap sample over all the subjects. The black horizontal bar highlights the stimulation period.

**Fig. 5:**
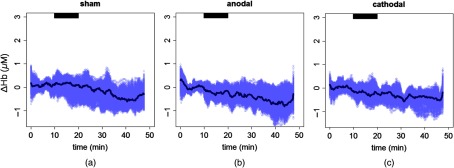
Deoxyhemoglobin (Hb) concentration during the three types of stimulation sessions on the left hemisphere from the fit analysis. (a) Sham session, (b) anodal session, and (c) cathodal session. The thick line is the average, and the lighter colored region represents the bootstrap sample over all the subjects. The black horizontal bar highlights the stimulation period.

For this reason, the fit analysis results are used for building the LME model for the changes in ${\mathrm{HbO}}_{2}$ and Hb concentrations. Furthermore, this is what is most commonly used for TR-fNIRS analysis.^29^ Figures 4 and 5 show the average time evolution and the bootstrap sample from all the subjects for ${\mathrm{HbO}}_{2}$ and Hb concentrations, respectively, in the left hemisphere during the three stimulation sessions.

As for the blood flow, among the effects considered in the LME model and listed in Sec. 2, only the stimulation improves the model. For both variables, the changes depend on the stimulation session but a recovery is not seen in the 30 min of post-periods, because the period is not a significant effect for the model.

**Fig. 6:**
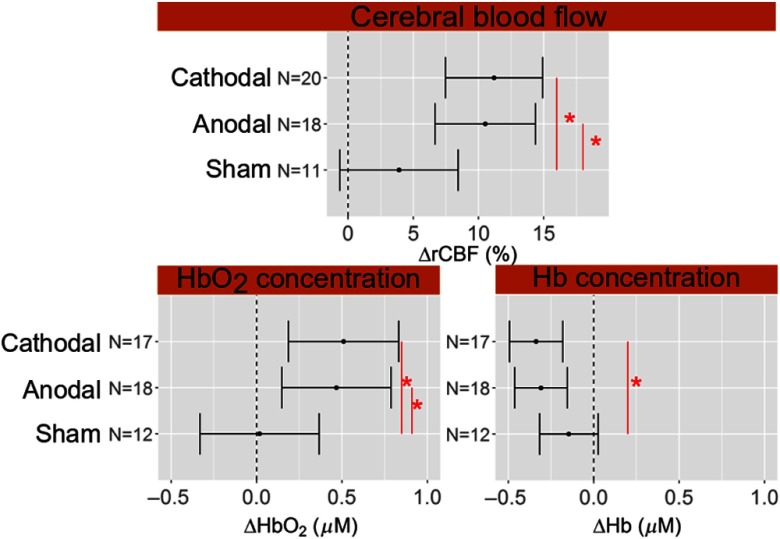
Changes of cerebral hemodynamics parameters during and after the stimulation sessions, as obtained by the LME model with the 95% confidence interval as the errorbars. N refers to the number of subjects used for the statistical analysis. Red asterisk highlights statistically significant difference between stimulation sessions. Dashed vertical line represents the zero level.

As summarized in Fig. 6, ${\mathrm{HbO}}_{2}$ concentration increased by 0.5  μM (CI: 0.1 to 0.8  μM) during the anodal session (p=0.002) and by 0.5  μM (CI: 0.2 to 0.8  μM) during the cathodal (p=0.001) session. As expected, the changes in Hb concentration were opposite to ${\mathrm{HbO}}_{2}$ concentration. Hb concentration decreased by −0.3  μM (CI: −0.5 to −0.1  μM) during the anodal session (p<0.001) and by −0.3  μM (CI: −0.5 to −0.2  μM) during the cathodal session (p<0.001). No change was seen in the sham session (p=0.1 for ${\mathrm{HbO}}_{2}$ and p=0.07 for Hb concentration). A difference was detected by our model for ${\mathrm{HbO}}_{2}$ concentration between anodal and sham (p=0.004) sessions, as well as between cathodal and sham (p=0.003) sessions. On the other hand, a pair comparison for Hb concentration gave a significant difference only between cathodal and sham sessions (p=0.03).

A frequency range from 1 to 20 Hz was considered for the EEG analysis. The lower limit was chosen because artifacts derived from movement and subsequent changes in the skin-electrode impedance affect mostly the low EEG frequencies. Furthermore, a time–frequency analysis of each recording was performed in order to study the signal quality of the dataset. In many recordings, the EEG spectrum was affected by external interferences in frequencies above 22 Hz. It was then decided to choose an upper cutoff frequency of 20 Hz to avoid this noise. As a result, the beta band covers 13 to 20 Hz.

In addition, after the temporal and spectral inspection of the EEG spectrum that was acquired during the stimulation, we have concluded that the quality was not sufficient to perform a reliable EEG analysis. Therefore, only recordings before and after the stimulation were used.

**Fig. 7:**
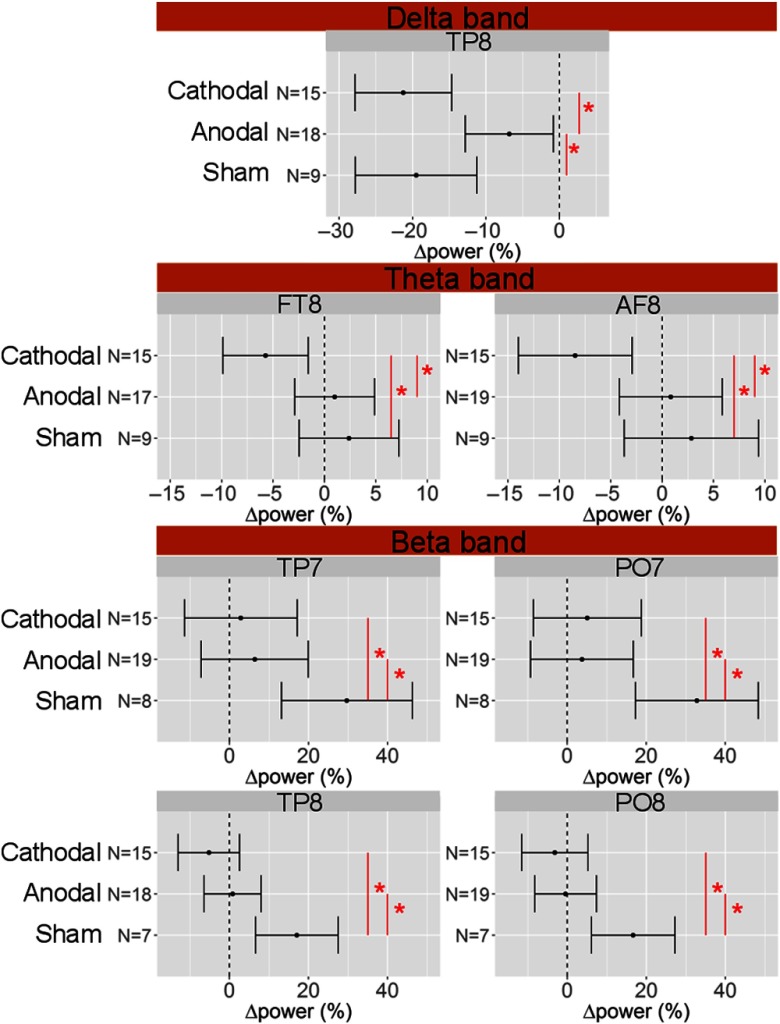
Change of EEG power in electrode TP8 in the delta band, electrodes AF8 and FT8 in the theta band, and electrodes TP7, PO7, TP8, PO8 in the beta band caused by the three types of stimulation, as obtained by the LME model, with 95% confidence interval as the errorbars. N refers to the number of subjects used for the statistical analysis. Red asterisk highlights the statistically significant difference between stimulation sessions. Dashed vertical line represents the zero level. Only bands and electrodes with statistical significant changes are shown.

Going through results obtained in EEG power, different changes from resting-state across stimulation modalities were observed only in the eyes-open condition. Figure 7 shows barplots for bands and electrodes where response was affected by the stimulation session according to LME model. Posthoc analysis resulted in a statistically significant power decrease in delta band in TP8 after every stimulation type. Statistically significant differences between anodal and cathodal sessions (p<0.001) and anodal and sham sessions (p=0.02) were observed. A statistically significant power decrease in the theta band after cathodal stimulation in fronto-right region (AF8, FT8) was observed. Statistically significant differences between cathodal and sham sessions (p=0.002 in both electrodes) and cathodal and anodal sessions (p=0.002 in both electrodes) were also observed. A more widespread effect in the beta band was observed. All electrodes in the tempo-parietal (TP7 and TP8) and parieto-occipital (PO7 and PO8) lobes experienced a statistically significant increase after sham, but, not after any active stimulation. In all these electrodes, the difference between anodal and sham sessions (p=0.004 for TP7, p=0.01 for TP8, p<0.001 for PO7, p=0.002 for PO8) and cathodal and sham sessions was significant (p<0.001 for all electrodes).

## 4 Discussion

In this work, we have introduced the use of fDCS, together with TR-fNIRS, as neuromonitors of cerebral hemodynamics during and after DC-tCS. The former has allowed the direct measurement of CBF primary biomarker of the neurovascular coupling. The measurement of cerebral hemodynamics was concurrent to EEG, which measures electrical activity. Therefore, information on both hemodynamics and neuronal activity from the same run of the protocol were available.

Before going through the results of the hemodynamics parameters, we note that the fDCS analysis requires the optical properties of the probed tissue as input parameters. In this experiment, the optical properties were measured by TR-fNIRS with fibers with a source–detector separation of 3.7 cm. On the other hand, source–detector separation of fDCS fibers was 2.5 cm, hence, probing a different volume than TR-fNIRS. Since our analysis assumes that the underlying tissue is homogeneous, we can tolerate this difference. It must also be highlighted that since we focus on the measurement of relative CBF, our results are relatively insensitive to small inaccuracies of the bulk optical properties.^56^

Statistical analysis confirms that CBF increases in the left ipsilateral hemisphere (in the region under the stimulation electrode) during anodal and cathodal sessions but not during the sham session. CBF does not recover during the 30 min following the stimulation. This is consistent with the fact that if the stimulation is applied for a period longer than 9 min, the effect of the stimulation persists long after the stimulation ends.^57^ Specifically, our statistical model results in a 10% increase during the anodal session and 11% during cathodal and gives no difference between the two stimulation conditions. This amount for CBF change is similar to what was assessed with arterial spin labeling, a magnetic resonance imaging technique,^58^ but, is much larger than what measured by positron emission tomography (PET).^59^ In Ref. 59, scans taken during the following hour after the stimulation were averaged, while we have measured only the following 30 min, which may account for some of the difference in our results. The optical probe placed in the right contralateral hemisphere did not detect any change in CBF in this region. The work done with PET^59^ allowed for whole brain imaging and detected regional increases in different areas of the brain, even far away from the stimulation electrodes, after both anodal and cathodal sessions compared to sham. Although anodal stimulation proved to cause a more widespread increase of regional CBF than cathodal stimulation, the same effect was seen for both stimulation types in the region under the electrode, which is consistent with our results.

It is known that diffuse optical techniques measure a mixture of extra- and intracerebral perfusion.^60^ This could be a confounding factor since DC-tCS causes a “skin redness” (erythema), which may lead to changes in the skin perfusion of different amounts between active and sham stimulations.^10^ However, the properties of photon diffusion into the tissue help us in explaining that response detected in this protocol is not mainly due to changes in skin perfusion due to the stimulation induced skin redness. The visitation probability of photons traveling into the tissue has a so-called “banana-shaped” pattern and touches the skin mainly in the region corresponding to the source and detector fibers.^61^ In our case, the fibers are separated by a distance of 2.5 cm while the electrode, placed in the middle, has a diameter of 1.2 cm. If we consider that various works^62^^,^^63^ have shown that the erythema is concentrated in the area under the electrode, our optical monitors are not particularly sensitive to this redness. Furthermore, a recent study^64^ measured the temperature under the electrode during the stimulation, detecting a nonsignificant change after 10 min of stimulation (our total time of stimulation). Only after 20 min of application, a significant increase of 1.36 °C was detected. However, we should account for the fact that this work used a larger electrode resulting in a lower current density than ours (0.06 versus $0.88\;{\mathrm{mA}/\mathrm{cm}}^{2}$). Interestingly, there is evidence suggesting that the current intensity and the electrode area are more important parameters leading to increased redness and discomfort due to the stimulation of more cutaneous receptors.^62^ In the above mentioned Ref. 64, temperature was measured using a current intensity of 2 mA, larger that what was used in our experiment. Therefore, we expect a minimal, nonsignificant temperature increase in our case. Another indication that the observed, long-term changes are not purely due to skin redness is that it is known that erythema disappears within 30 min after the stimulation.^65^ In our case, the stimulation response did not decrease after 30 min.

Proceeding to TR-fNIRS measurements, results from the different analysis methods considered were compared. To start with, DPF analysis gives us the results that would have been obtained using a typical CW-fNIRS setup for fNIRS, the most commonly used fNIRS technology. DPF analysis shows an increase of about 2  μM in ${\mathrm{HbO}}_{2}$ concentration in the left hemisphere compatible with what was previously found in the literature.^20^^,^^22^ This increase is seen in active and sham stimulations, where the brain is not actually stimulated which questions its validity. Focusing on the results given by the analysis of variance, the second moment of the DTOF, we retrieve smaller changes in both anodal and sham sessions with respect to DPF analysis. Notably, the variance of TR-fNIRS curves is more sensitive to the deeper layers with respect to the intensity, the parameter used in the DPF analysis.^48^ This may suggest that results in DPF analysis are contaminated by superficial effects present both during anodal and sham stimulations. Moreover, these results are in accordance with a previous single subject experiment, where TR-fNIRS was used during anodal stimulation.^21^ In this work, TR-fNIRS measurements were analyzed with a gated analysis.^66^ This method exploits the depth information encoded in time in the broadened pulse collected after propagation into the tissue and allows to decouple changes in the extracerebral and cerebral layers. Measurements performed on a single subject during anodal stimulation retrieved a larger ${\mathrm{HbO}}_{2}$ concentration change in the superficial than in the deeper layer, which would be heavily reflected in the DPF analysis. We would like to point out that the time gating analysis of TR-fNIRS was not implemented here because the lack of knowledge of the extracerebral layer thickness on an individual basis compromises the validity of this method.^67^

The usage of TR-fNIRS has allowed us to improve the separation of the extracranial and intracranial signals for the analysis of the hemoglobin concentration changes. We do not have a comparable time-domain measurement for fDCS at the moment.^68^^,^^69^ However, it has been proved that the relative brain-to-scalp sensitivity is about three times higher for fDCS than for CW-fNIRS.^70^ Therefore, we believe that our results are reflective of the CBF in a more reliable manner than the CW-fNIRS results.

Since the fit analysis gives results comparable to the variance, and this is the most commonly used method when measuring oxygenation with TR-fNIRS, we have decided to consider the results given by the fit analysis.

In this protocol, ${\mathrm{HbO}}_{2}$ concentration increases and Hb concentration decreases in the left hemisphere during both anodal and cathodal stimulations, but not during sham. The right frontal lobe (contralateral to the stimulation) does not present changes in the oxygenation as is also true for CBF. Even if changes retrieved were very small, less than 1  μM, ${\mathrm{HbO}}_{2}$ and Hb concentrations can mark the status of the ongoing stimulation. As a matter of fact, the changes are statistically significant during both anodal and cathodal sessions but not during the sham session. ${\mathrm{HbO}}_{2}$ concentration is a more robust marker, since response to anodal and to sham sessions is statistically significantly different, as well as the response to the cathodal and to sham sessions. On the other hand, for Hb concentration, the contrast-to-noise ratio is not enough to give a statistically significant difference between anodal and sham sessions, but a decrease is not seen in the sham session.

The directions of Hb and ${\mathrm{HbO}}_{2}$ concentration changes are consistent with what is expected in the case of a cerebral activation and what was previously found in the literature.^20^^,^^22^ Specifically, due to the neurovascular coupling, when a cerebral region is activated, CBF is recruited to meet the additional demand of oxygen and it increases.^17^ Concurrent to this, an increase of ${\mathrm{HbO}}_{2}$ concentration and a decrease of Hb concentration are usually detected.^71^ This suggests that the brain is over supplied by oxygen by the increase of CBF compared to the oxygen consumption. The details of this process are, in fact, more complex.^17^ The time series of the hemodynamics parameters in Figs. 2, 4, and 5 suggest a steeper increase of CBF than the ${\mathrm{HbO}}_{2}$ and Hb concentrations. The above-mentioned mechanism of neurovascular coupling can explain this.^71^ Right after the stimulus, the supply of CBF does not overcome the need immediately. Only after this happens, a ${\mathrm{HbO}}_{2}$ concentration increase and Hb decrease is detected. This process was previously verified during *in vivo* protocols.^72^ Nonetheless, to make quantitative inferences on the time lag, we should have had a better time resolution than what we have here.

Both the anodal and cathodal stimulation sessions have created similar changes in the hemodynamics parameters. All the reviewed and cited published works^58^^,^^59^ that measure cerebral hemodynamics during DC-tCS show the same pattern for both cathodal and anodal stimulations in the region under the electrode. Our results confirm these findings. In addition, PET studies concurrent to transcranial magnetic stimulation also demonstrate an increase in CBF in the region under the stimulation for both excitatory and inhibitory protocols.^73^ These results suggest that CBF is not a marker of the polarity of the stimulation, at least in the directly stimulated area, and that regardless of whether the stimulation results in an activation of inhibitory or excitatory synapses, it leads to an increase in blood flow. It has been highlighted that this behavior of CBF reflects the local levels of synaptic activity in intracortical neurons and inputs to those areas rather than the activity of output neurons, which is opposite when the stimulation is excitatory or inhibitory.^59^^,^^74^

The stimulation protocol used in this study influenced EEG rhythmic activity at different bands and electrodes. Comparing the results from previously published studies about DC-tCS on the frontal cortex is not a trivial task since EEG response depends heavily on the electrode montage.^75^ All of the previous studies used F3 as the stimulation electrode while the return electrode, here referring to the electrode not placed in the frontal cortex with opposite polarity, was placed either on the supraorbital region or on extracerebral regions. By contrast, we have decided to use the AF7 electrode as stimulation electrode because the optical measurement series on the frontal brain region are more reliable due to the absence of hair. The return electrode was chosen to be spatially and functionally distant from the stimulation electrode in order to minimize the interactions and to stimulate the frontal region unilaterally.

Moreover, few studies measured the EEG spectrum at rest while most of them assessed the response to a task and how this can be influenced by the stimulation. It must be noted that a recent systematic review highlighted that results in the EEG spectrum of the same protocol were not consistent within different works.^76^ They concluded that there is no evidence that the EEG spectrum is affected by the stimulation. It may also be hypothesized that, since it is not trivial to maintain subjects in a well-controlled condition, the variability of the effects is increased.

We note that the protocol is 50 min long, which may have led to a sense of drowsiness and/or fatigue in the subjects, which could alter the cerebral electrophysiology and metabolism. The staring and attention tasks were implemented to minimize this effect. Furthermore, the transition from eyes open and closed and vice versa also helped with this issue. In spite of this, we cannot exclude that some of the subjects may have been affected and we do not have any control over this.

In order to define EEG spectrum results, effects in different frequency bands have been studied since different bands are traditionally related to different brain functions. It is, however, controversial whether the activity at a certain frequency band is related to a single brain function or that complex stimuli are reflected on a single oscillatory activity.^77^ This may explain the multiplicity of bands affected by our protocol.

The protocol applied in this study influenced brain activity in the delta, theta, and beta bands. No effect was detected in the alpha band, which is consistent with previous works.^76^

We will focus, from now on, on the bands, where the stimulation has proven to be effective. The changes in the delta band were observed in the right tempo-parietal region after all stimulation sessions but were significantly smaller in the anodal session compared to the other two. On the other hand, changes in the theta band occurred in the right fronto-temporal area exclusively after cathodal stimulation. Interestingly, delta and theta bands are associated with functions linked to the frontal cortex, which was stimulated in our protocol. It is an indication of activity in cognitive and memory performances,^78^ attention,^79^ and working memory.^80^ A response in these bands is consistent with previous work. Specifically, a lower power in delta and beta bands after the stimulation compared to sham has been previously observed,^35^^,^^81^ where pre- and post-stimulation spectral power was not calculated but the comparison between stimulation sessions was done in absolute values. This effect has been found only in the frontal cortex, in contrast to our findings, but both stimulation and return electrodes were placed on the anterior part of the head, while we have an electric field crossing all the brain. A significant and selective decrease of the power of theta band in the right inferior frontal gyrus area has been reported after DC-tCS stimulation along with an improvement in behavioral inhibition.^82^

Differences in EEG power across different stimulation sessions were also seen in the beta band. Power increased after the sham session in temporal and parietal regions, but not after active stimulation sessions. An unchanged or a decreased level of beta activity is usually observed in tasks where behavior is driven by bottom-up signals, while enhancement occurs if the system has to actively maintain endogenous attention.^83^ It may be hypothesized that the detected increase in beta activity is linked to an active effort to maintain the cognitive concentration state required for the sustained attentional task along the experimental protocol. This activity was present during sham but not during active stimulation sessions. Several references highlight an influence of DC-tCS when applied on the frontal cortex, on sustained attention.^84^ In our protocols, the same effects for anodal and cathodal sessions were found in the EEG beta band in the posterior part of the brain, without a differentiation due to the polarity. Intuitively, this is not expected because opposite polarity stimulation should produce an opposite electric field in the brain, i.e., either excitatory or inhibitory. Nonetheless, it must be mentioned that the predominance of excitatory or inhibitory electric field in an area of the brain does not exclude that some “pockets” of the opposite direction are present.^85^ We can speculate that only excitatory areas influenced the power in the beta band in the posterior area of the brain in our protocol. Unfortunately, this is prohibitively hard to confirm since not only the electrode montage but also the concurrent tasks alter the response to a certain polarity of stimulation,^86^^,^^87^ i.e., it would require a special protocol to address this. For our protocol, the attention task was not the primary goal.

Last, it was verified whether the change in CBF in the left frontal cortex due to stimulation has any correlation with the power change respect to baseline in any of the EEG bands and electrodes. This was done even for the electrodes far away from the optical probes since the source of the EEG signal can lay far from the scalp point, where it is registered and its exact location cannot be well determined due to the inverse problem.^88^ The Pearson correlation coefficient was calculated and a correlation was defined significant if, by bootstrapping the sample (i.e., repeating the analysis removing one subject at time), the correlation stays significant. After this analysis, we have found a negative correlation in the delta band for electrode FT8 with a Pearson correlation coefficient of R=−0.6. This is consistent with a predicted and verified decrease in lower frequency band during activation.^89^^,^^90^ The study of the correlation between the hemodynamics and neuronal activity is a very appealing topic due to the important information on the neurovascular coupling it could provide. It is also a very complicated process since several factors can contribute to the failure of the correlation between EEG activity and hemodynamics.^91^ A detailed characterization of this is beyond the scope for our paper, which aimed at the introduction of hybrid diffuse optical devices for monitoring the response to the stimulation, and requires a dedicated protocol with more regions probed for hemodynamics.

In summary, we have introduced fDCS as a neuromonitoring technique for following the response to DC-tCS. It was integrated with TR-fNIRS to measure the concentrations of ${\mathrm{HbO}}_{2}$ and Hb in a more accurate way with respect to CW-fNIRS. We have proved that fDCS and TR-fNIRS can be applied concurrently to EEG measurements, which assess cerebral activity. We were able to have information on both cerebral hemodynamics and neuronal activity in the same run of stimulation.

## 5 Conclusion

We have shown that fDCS and TR-fNIRS are suitable tools to monitor cerebral hemodynamics during and after DC-tCS and they can be integrated with EEG, which monitors the neuronal activity. We have found CBF to be a good indicator of the ongoing stimulation since it showed an increase during and after anodal and cathodal stimulation in the region under the stimulation electrode. Results obtained with TR-fNIRS showed small changes of ${\mathrm{HbO}}_{2}$ and Hb concentrations but the effect of the stimulation was still detectable.

By concurrent EEG recordings, we could follow the modulation of the underlying neural oscillations. DC-tCS over the frontal cortex induced statistically significant power changes across different stimulation sessions in delta, theta, and beta EEG rhythms.
